# The Influence of Age on the Pharmacokinetics of Enrofloxacin and Ciprofloxacin in Calves

**DOI:** 10.3390/vetsci13060538

**Published:** 2026-05-29

**Authors:** Orhan Corum, Murat Yuksel, Duygu Durna Corum, Elisa Escudero, Pedro Marín, Mario Giorgi, Halis Oguz, Erdinc Turk, Devran Coskun, Elena Badillo, María Teresa Yuste, Kamil Uney

**Affiliations:** 1 Department of Pharmacology and Toxicology, Faculty of Veterinary Medicine, University of Hatay Mustafa Kemal, Hatay 31060, Türkiye; orhan.corum@mku.edu.tr (O.C.); duygu.durnacorum@mku.edu.tr (D.D.C.); erdincturk@mku.edu.tr (E.T.); 2 Department of Obstetrics and Gyneacology, Faculty of Veterinary Medicine, University of Hatay Mustafa Kemal, Hatay 31060, Türkiye; murat.yuksel@mku.edu.tr; 3 Department of Pharmacology, Faculty of Veterinary Medicine, University of Murcia, 30100 Murcia, Spain; escudero@um.es (E.E.); pmarin@um.es (P.M.); ebp2@um.es (E.B.); mariateresa.yuste1@um.es (M.T.Y.); 4 Department of Veterinary Sciences, University of Pisa, San Piero a Grado, 56121 Pisa, Italy; mario.giorgi@unipi.it; 5 Department of Pharmacology and Toxicology, Faculty of Veterinary Medicine, University of Selcuk, Konya 42031, Türkiye; halisoguz@selcuk.edu.tr; 6 Department of Pharmacology and Toxicology, Faculty of Veterinary Medicine, University of Siirt, Siirt 56100, Türkiye; devran.coskun@siirt.edu.tr

**Keywords:** age, cattle, ciprofloxacin, enrofloxacin, intravenous, pharmacokinetics

## Abstract

This study investigated age-related variations in the pharmacokinetics of enrofloxacin and its active metabolite, ciprofloxacin, in calves. This study was carried out on a total of 18 calves using a parallel pharmacokinetic design. Eighteen animals were assigned to three age groups, one, four, and eight months old, and received a single intravenous dose of 10 mg/kg enrofloxacin. Serial blood samples collected over 48 h were analyzed using high-performance liquid chromatography to determine plasma concentrations. In one-month-old calves, the pharmacokinetic parameters revealed a volume of distribution at steady state (V_dss_) of 1.44 L/kg, total clearance (Cl_T_) of 0.42 L/h/kg, elimination half-life (t_1/2ʎz_) of 2.52 h, and area under the concentration versus time curve (AUC_0-last_) of 23.36 h*μg/mL. Notably, the AUC_0-last_ of enrofloxacin was increased and Cl_T_ decreased in the older age groups (four and eight months). Additionally, the V_dss_ of enrofloxacin significantly decreased in eight-month-old calves relative to younger groups, while t_1/2ʎz_ showed statistically significant differences in the order of four months > eight months > one month. The conversion ratio of enrofloxacin to ciprofloxacin was highest in one-month-old calves (43.39%) and diminished in four-month-old (25.73%) and eight-month-old (23.04%) calves. The results indicated that the pharmacokinetics of enrofloxacin and ciprofloxacin at a dose of 10 mg/kg in cattle exhibited significant variation with age. However, pharmacokinetic/pharmacodynamic investigations in cattle of different ages are required to assess the impact of these pharmacokinetic alterations on clinical efficacy.

## Simple Summary

This study examined the pharmacokinetic behavior of enrofloxacin and its active metabolite, ciprofloxacin, in calves at various developmental stages. Three age groups (one, four, and eight months old) were evaluated to assess how age influences drug disposition. This research focused on comparing key pharmacokinetic parameters such as distribution volume, clearance, half-life, and the metabolic conversion ratios to determine age-related differences. The results highlighted significant variations in drug pharmacokinetics among different age groups, with younger calves showing a higher metabolic conversion ratio and distinct pharmacokinetic profiles. These findings emphasize the value of considering age when determining appropriate dosing regimens for enrofloxacin in calves. This study provides valuable insights into how growth and maturation affect enrofloxacin pharmacokinetics, which can inform more effective and safer therapeutic strategies in veterinary practice.

## 1. Introduction

Fluoroquinolones, developed by adding a fluorine atom to nalidixic acid, the first member of the quinolones, are extensively used in human and veterinary medicine [1]. Fluoroquinolones are categorized as category B (restricted) by the European Medicines Agency and are suggested for the treatment of clinical diseases without viable alternative antibiotics in a lower (C and D) category [2]. These drugs are widely used because of their large volume of distribution, resulting in higher tissue and fluid concentrations than plasma concentrations; low minimum inhibitory concentrations for susceptible bacteria; rapid onset of action; and few side effects [3].

Enrofloxacin (ENR) is a fluoroquinolone-class antibiotic developed for use in animals. This antibiotic has high lipophilicity, and the presence of a carboxylic acid (pKa = 5.88–6.06) and a tertiary amine group (pKa = 7.70–7.74) in its structure imparts amphoteric characteristics [1,4]. It shows a bactericidal effect by inhibiting bacterial DNA topoisomerase II (gyrase) and DNA topoisomerase IV enzymes [1]. It is deethylated to form ciprofloxacin (CIP), a metabolite whose antimicrobial mechanism of action and activity are similar to the parent drug [5]. ENR and CIP exhibit a wide range of efficacy against Gram-positive and Gram-negative bacteria, as well as *Mycoplasma* species [6]. ENR is intended for use in cattle for respiratory and gastrointestinal infections, mastitis, septicemia, and *Mycoplasma*-related arthritis [1,7].

Many physiological changes occur in mammals that are age-dependent. The body’s water/fat ratio, blood components, plasma protein content, liver and kidney functions, and enzyme capability all alter with age [8,9]. These physiological alterations may modify the pharmacokinetics and hence the therapeutic efficacy of drugs in animals of varying ages [10]. Failure of antibiotic treatment, which is shaped by pharmacokinetic changes, can lead to disease-related losses and especially the development of antimicrobial resistance [11].

Bacterial infections are common in cattle of all age groups and cause significant productivity losses and mortality [12]. ENR is administered in similar doses in the treatment of bacterial infections in cattle of all age groups [7,13]. It has previously been reported that the pharmacokinetics of ENR and its active metabolite CIP in premature calves (1-day-old vs. 1-week-old calves) change with age [5,14]. In addition, age-related changes in the pharmacokinetics of ENR and CIP have been reported in other mammalian and bird species [11,15,16,17,18]. As the antibacterial effect of ENR is concentration-dependent, changes in its plasma concentrations may alter its therapeutic efficacy [19]. Therefore, determining the pharmacokinetic properties of ENR in target age groups is critical to strengthen antimicrobial stewardship and reduce the development of resistance. It was hypothesized that physiological changes occurring as a result of maturation in cattle may alter the pharmacokinetics of ENR and CIP by affecting their distribution, metabolism, and excretion. The aim of this study was to compare the pharmacokinetics of ENR and its active metabolite, CIP, after the intravenous administration of ENR at a dose of 10 mg/kg to one-, four-, and eight-month-old calves.

## 2. Materials and Methods

### 2.1. Chemicals

The analytical standards for ENR (≥99%) and CIP (≥98%) were obtained from Sigma-Aldrich (St. Louis, MO, USA). HPLC-grade chemicals were used for the analysis of ENR and CIP. Acetonitrile was acquired from Chemlab (Zedelgem, Belgium), while triethylamine and orthophosphoric acid were obtained from Merck (Darmstadt, Germany). The injectable formulation of ENR (Baytril, 100 mg/mL, Bayer, Istanbul/Türkiye) was used for drug administration to calves.

### 2.2. Animals

This research was conducted on eighteen Holstein calves on a private farm, and the experimental procedure was approved (2025/02-04) by the Hatay Mustafa Kemal University Animal Research Local Ethics Committee. Calves were selected from 1-month (n = 6, 40–55 kg), 4-month (n = 6, 120–140 kg), and 8-month (n = 6, 260–300 kg) age groups to represent various age categories. This study included calves that had not received any medication in the preceding 15 days and were considered healthy based on clinical evaluations. The one-month-old calves were kept in individual pens, and calves of other age groups were kept in paddocks. The calves were fed age-appropriate commercial feed, and hay and water were kept available at all times. In addition, one-month-old calves were given breast milk. Ear tags and numbered halters were used to facilitate the identification of calves. The experimental study was conducted over the same time period for all age groups.

### 2.3. Experimental Design

Eighteen calves were divided into three equal groups: one month (n = 6), four months (n = 6), and eight months old (n = 6). The animals were weighed for drug administration. ENR was administered intravenously (left jugular vein) at a dose of 10 mg/kg to all age groups. Blood collection (1–2 mL) was carried out at 15 different sampling times: 0, 0.08, 0.25, 0.5, 0.75, 1, 2, 4, 6, 8, 10, 12, 18, 24, and 48 h. Blood samples were collected using a catheter (22G, 0.9 × 25 mm) inserted into the right jugular vein for the first 12 h and by venipuncture from both jugular veins at other times. The samples were immediately placed in tubes containing lithium heparin, shaken gently several times, and stored in cold boxes. Blood samples were centrifuged (4000× *g* for 10 min) within 1 h. The plasma samples were transferred to microcentrifuge tubes and stored at −80 °C until analysis.

### 2.4. HPLC Conditions

The plasma concentrations of ENR and CIP were quantified using high-performance liquid chromatography (HPLC) with ultraviolet detection, the method previously reported [14]. The HPLC system (Shimadzu, Tokyo, Japan) was equipped with a model LC-20AT pump, a model SIL 20A auto-sampler, a model CTO-10A column oven, a model DGU-20A degasser, and a model SPD-20A UV detector. The Gemini^TM^ C18 column (4.6 × 250 mm; 5 μm, GL Sciences, Tokyo, Japan) was used for the chromatographic separation of ENR and CIP. The quantification wavelength was established to be 280 nm. The column oven was set at 40 °C. Acetonitrile (18%) and a mixture of (0.4%) orthophosphoric acid with (0.4%) triethylamine (82%) were used for the mobile phase. The flow rate was set at 1 mL/min. Data analysis was performed using the LC Solution program (Version 1.25 SP5) from Shimadzu Corp.

### 2.5. Method Validation

The chromatographic process was validated in accordance with EMA criteria [20]. ENR and CIP stock solutions were prepared in 0.01 M NaOH at a concentration of 1 mg/mL. To generate working standards for ENR (0.04–40 μg/mL) and CIP (0.04–4 μg/mL), the stock solution was diluted with ultrapure water. Calibration standards were prepared by adding working standards to blank calf plasma. Calibration standards were in the range of 0.04–40 μg/mL for ENR and 0.04–4 μg/mL for CIP, and the low (0.1 μg/mL for CIP and ENR), medium (1 μg/mL for CIP and ENR) and high (2 μg/mL for CIP and 20 μg/mL for ENR) levels of these values were used as quality control samples. The evaluation of quality control samples was performed six times on five separate days to ascertain recovery, precision, and accuracy. Recovery was assessed by comparing the peak areas of quality control concentrations to those of working standards at the same concentrations. Precision was evaluated using the coefficient of variation, whereas accuracy was determined as bias.

### 2.6. Sample Extraction

Plasma samples were allowed to come to room temperature. In total, 200 μL of calf plasma was transferred to a microcentrifuge tube, and 400 μL of acetonitrile was added to denature the proteins. The mixture was vortexed for 30 s and then centrifuged at 12,000× *g* for 10 min. Then, 100 μL of supernatant was taken and transferred to a new microcentrifuge tube, and 100 μL of ultrapure water was added. This mixture was vortexed for 10 s, then transferred to autosampler vials, and 20 μL was injected into the system. CIP and ENR eluted at approximately 6.5 and 8.5 min, respectively, with a total run time of 19 min.

### 2.7. Pharmacokinetic Analysis

The chromatogram data from HPLC analysis for ENR and CIP were recorded in an Excel file, plasma concentrations were calculated, and plasma concentration–time curves were plotted. The pharmacokinetic parameters of ENR and CIP for each calf were assessed by non-compartmental analysis utilizing WinNonlin 6.1.0.173 software (Pharsight Corp., Mountain View, CA, USA). The abbreviations and definitions of pharmacokinetic parameters are provided in the footnote. For ENR, t_1/2ʎz_, MRT, V_dss_, Cl_T_, AUC, and AUC_extrap%_ values were calculated. The AUC, AUC_extrap%_, C_max_, and T_max_ values were calculated for CIP. The C_max_ and T_max_ were determined by a direct examination of the plasma concentration–time curve. The conversion ratio of ENR to CIP in different age groups was calculated with the following formula (AUC_CIP_/AUC_ENR_ ∗ 100).

### 2.8. Pharmacokinetic/Pharmacodynamic Integration

The pharmacokinetic/pharmacodynamic integration was calculated using pharmacokinetic data obtained in this study and previously reported minimum inhibitory concentration (MIC) values. ENR shows a concentration-dependent effect, and AUC/MIC and C_max_/MIC data were used to evaluate its antibacterial activity [19]. However, since ENR was administered intravenously in this study, only the AUC/MIC parameter was calculated. The AUC value obtained from the free fraction of the drug must be used for this calculation. Because plasma protein binding was not determined in this study, the free fraction was calculated by averaging previously reported binding ratios (40–61%) in cattle [21,22].

### 2.9. Statistical Analysis

The pharmacokinetic parameters were presented as the geometric mean (min–max) except for T_max_, which was displayed as the median (min–max). The statistical evaluation was carried out using the SPSS 22.0 program, and a *p*-value of 0.05 was accepted as statistical significance. The Shapiro–Wilk test was used to assess data normality, whereas the Levene test was used to assess homogeneity. The one-way analysis of variance (ANOVA) and post hoc Tukey test were used to compare differences in pharmacokinetic parameters between age groups.

## 3. Results

### 3.1. Safety

The IV injection of ENR at a dose of 10 mg/kg to calves aged one month, four months, and eight months did not result in any local or systemic adverse effects. The behavior, feed and water intake, and fecal production of all calves were normal.

### 3.2. Method Validation

The working and calibration curves exhibited linearity with an R^2^ value of >0.997 for ENR and >0.991 for CIP. The mean recovery in calf plasma was greater than 93% for ENR and 89% for CIP. For ENR and CIP in calf plasma, the limit of detection and lower limit of quantitation were 0.02 and 0.04 μg/mL, respectively. The intra- and inter-day coefficients of variation for ENR and CIP were below 9.26% and 7.89%, respectively. The intra- and inter-day biases for ENR and CIP were in the range of ±6.98% and ±5.62%, respectively.

### 3.3. Pharmacokinetic Parameters of Enrofloxacin

**Figure 1:**
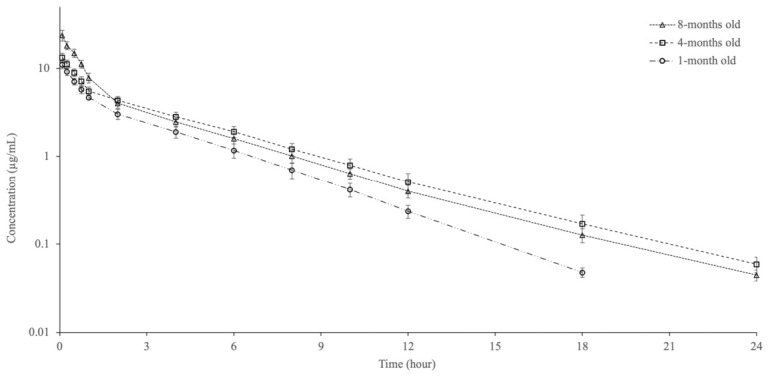
Semi-logarithmic plasma concentration–time curves of enrofloxacin (10 mg/kg) following intravenous administration in one-, four- and eight-month-old calves (n = 6).

Figure 1 illustrates the plasma concentration–time profiles for ENR in calves of different ages. ENR was detected in plasma up to 18 h in one-month-olds and up to 24 h in other ages. The pharmacokinetic parameters of ENR in calves of different ages are presented in Table 1. The AUC was elevated and the Cl_T_ was diminished in calves aged 4 and 8 months compared to those aged 1 month (*p* < 0.05). The V_dss_ was lower in 8-month-old calves than in other age groups (*p* < 0.05). The t_1/2ʎz_ showed significant differences between groups in the order of four months > eight months > one month (*p* < 0.05). The AUC_extrap_ was below 1.2% in all groups.

### 3.4. Pharmacokinetic Parameters of Ciprofloxacin

**Figure 2:**
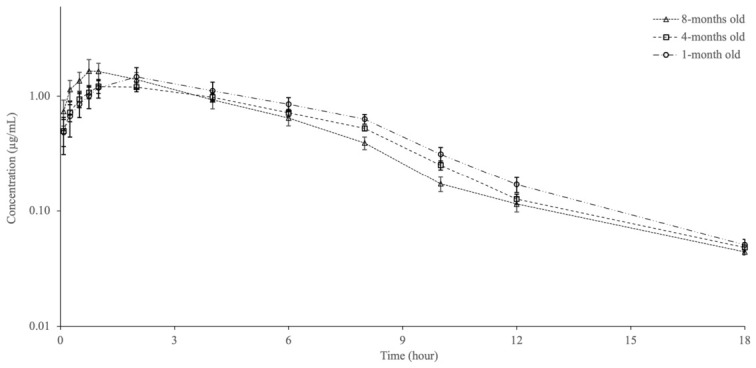
Semi-logarithmic plasma concentration–time curves of ciprofloxacin following intravenous administration of enrofloxacin (10 mg/kg) in one-, four- and eight-month-old calves (n = 6).

The plasma concentration–time profiles and pharmacokinetic data of CIP following the IV administration of ENR in calves of different ages are presented in Figure 2 and Table 1, respectively. CIP was detected up to 18 h in one-, four-, and eight-month-old calves. The AUC values were similar in all age groups (*p* > 0.05). The C_max_ was higher at eight months of age compared to four months of age, while T_max_ was longer at one month of age (*p* < 0.05). The AUC_0-∞CIP_/AUC_0-∞ENR_ ratio was higher in the one-month age group (*p* < 0.05). The AUC_extrap_ was below 3% in all groups.

## 4. Discussion

Drug plasma concentrations may vary due to age-related pharmacokinetic changes [5,11]. These changes in plasma concentration levels may affect the therapeutic efficacy of antibiotics that have a concentration-dependent effect, such as ENR, and therefore the success of treatment. The pharmacokinetics of ENR and CIP differed in calves of different ages (premature, 1 day old, and 1 week old) [5,14], lactating dairy cows, and beef cattle [21]. Thus, it is critical to conduct pharmacokinetic studies of drugs in the target age group. ENR is used at the same doses in bacterial infections caused by susceptible bacteria in cattle of all age groups [7,13]. It might not be suitable to use the same dosage regimen of ENR across all age groups due to pharmacokinetic differences. The pharmacokinetics of ENR and its active metabolite CIP were studied in calves aged one, four, and eight months for the first time, revealing significant variations among the age groups.

The administration of ENR at a dose of 10 mg/kg to calves at one, four, and eight months of age exhibited no adverse effects. It is suggested that ENR be administered to cattle at a dose of 2.5–5 mg/kg for a duration of 3 days or 7.5–12.5 mg/kg as a single dose [13]. Previous pharmacokinetic studies in cattle utilized dosages of 2.5 mg/kg and 12.5 mg/kg of ENR [5,23]. As ENR is a concentration-dependent drug, it has been suggested to administer high doses at a time for better efficacy in calves [13]. Therefore, the 10 mg/kg dose was preferred in this study. The IV administration was chosen as it is one of the recommended administration routes in ENR treatment and allows for the assessment of alterations in Cl_T_ and V_d_ parameters.

ENR has a generally wide V_dss_ (1.40–7.30 L/kg) [24,25] due to its lipophilic structure and its moderate binding to plasma proteins (40–61%) in cattle [21,25]. V_dss_ and V_darea_ are the volumes in steady-state and pseudo-equilibrium, respectively [26]. In this study, the closeness of V_dss_ (0.88–1.44 L/kg) and V_darea_ (1.14–1.54 L/kg) to each other indicates that a minimum amount of ENR is eliminated in the distribution phase. V_dss_ is an indicator of the distribution of the drug into body tissues, and a high value indicates that the ENR is well distributed into extravascular tissues. Studies show that tissue and fluid concentrations of ENR are higher than plasma concentrations [27,28]. The V_dss_ value of ENR was different in one-month-old (1.44 L/kg), four-month-old (1.27 L/kg), and eight-month-old cattle (0.88 L/kg), and this value was observed to decrease with age. While the V_dss_ was larger in 1-week-old calves than in 1-day-old calves [5], it was similar in lactating dairy cows and beef steers [21]. The V_dss_ was larger in 1-month-old sheep than in 6- or 12-month-old sheep [11]. The results indicate that age-related alterations in V_dss_ vary between studies. V_dss_ is affected by changes in the plasma protein binding ratio and body fat–water composition [29]. The plasma protein binding ratio of ENR was similar in dairy cows (body weight: 584–727 kg, 60%) and beef cattle (body weight: 159–213 kg, 61%) [21]. Furthermore, the plasma protein binding ratio was determined to be age-independent in turkeys [18] and rabbits [17]. Therefore, the age-related change in V_dss_ may not be due to changes in plasma protein binding. In cattle, the V_d_ of amphoteric drugs such as norfloxacin is reported to be greater in young animals than in adults due to the decrease in total body water and increase in fat with age [30]. Fluoroquinolones bind to the articular cartilage of young animals and accumulate in high amounts in this area [30,31]. In addition, the rumen constitutes a significant part of the body part in ruminants, and rumen development and content volume vary depending on age [32]. Since tissue distribution and plasma protein binding were not measured in this study, it can only be suggested that the high V_dss_ observed in one-month-old cattle might be related to the accumulation of ENR in the joint cartilage and differences in body composition.

ENR is converted into metabolites after undergoing various biotransformation reactions, and the most important active metabolite is CIP [4]. Enrofloxacin is excreted primarily by the kidney via glomerular filtration and tubular secretion and is also excreted in the feces [33,34]. The Cl_T_ of ENR was faster in one-month-old calves than in four- and eight-month-old calves. Previous studies showed that Cl_T_ of ENR was higher in 1-week-old calves than in 1-day-old calves [5] and in lactating dairy cows than in beef cattle [21]. In addition, this value was similar in one-, six-, and twelve-month-old sheep [11]. The CIP conversion ratio in one-, four-, and eight-month-old calves was between 23 and 43%, indicating that biotransformation is important in the elimination of ENR in calves. ENR is metabolically transformed into CIP through P450-dependent deethylation [5]. Liver microsomal enzyme levels and activities change depending on age. Hepatic enzyme activity and CYP450 content reach adult levels in 6 weeks in pigs and 8–10 weeks in goats [35]. In 42-day-old calves, the amount of hepatic microsomal protein is similar to adults, while CYP enzyme activity is low. It was also stated that biotransformation and excretion pathways in 6-week-old Holstein calves may be at adult levels [36]. Renal functions such as glomerular filtration and tubular secretion of ruminants reach mature levels within 1–2 weeks [30,37]. However, urine pH is influenced by the diet of calves, which varies based on their age [30,37]. Moreover, changes in urine pH influence the tubular reabsorption of the fluoroquinolones [37,38]. As liver enzyme activity and renal functions were not directly evaluated, the high Cl_T_ in one-month-old calves can only be interpreted as possibly resulting from variations in biotransformation enzyme levels and activity, as well as differences in excretion pathways and diets.

The t_1/2ʎz_ showed significant differences between groups. The longest and shortest values for t_1/2ʎz_ were obtained for 4-month-old and 1-month-old calves, respectively. It has been reported that t_1/2ʎz_ of ENR is longer in 1-day-old calves than in 1-week-old calves and in beef steers than in lactating dairy cows [5,21]. The t_1/2ʎz_ is a hybrid parameter consisting of Cl_T_ and V_d_ values and is inversely proportional to Cl_T_ and directly proportional to V_d_ [39]. The difference in t_1/2ʎz_ in one-, four-, and eight-month-old calves may be due to the age-related changes in Cl_T_ and V_dss_.

ENR undergoes deethylation via the P450 system in several animal species, resulting in the pharmacologically active metabolite CIP [4]. The AUC_0-∞CIP_/AUC_0-∞ENR_ ratio in cattle was documented to range from 18% to 64% [5,21]. The AUC_0-∞CIP_/AUC_0-∞ENR_ ratios were 43.39%, 25.73%, and 23.30% for calves aged one, four, and eight months, respectively. These values were consistent with those reported in the aforementioned studies. The variations in CIP conversion ratios in cattle may result from changes in age, body weight, breed, physiological stages (lactation, etc.), dose, and analysis methods [11,23]. The AUC_0-∞CIP_/AUC_0-∞ENR_ ratio was higher in 1-month-old calves than in 4-month-old and 8-month-old calves. Although it is unknown which enzymes are involved in the conversion of ENR to CIP, some authors’ reports suggest that the CYP3A4 enzyme may play an important role in pigs [40]. Although there is no information about CYP3A4 enzyme activity in calves of different ages, it has been reported that the amount of this enzyme is higher in 4-week-old sheep than in 7- and 11-month-old sheep [41]. While CIP was detected in plasma up to 18 h in all age groups, ENR was detected up to 18 h in one-month-old calves and up to 24 h in other ages. This difference in the plasma concentration–time curve affects the AUC value. The AUC value for CIP was consistent across all groups; however, it was lower for ENR at one month of age (*p* < 0.05). Based on these findings, it can be inferred that the higher conversion ratio of ENR to CIP in 1-month-old calves may be attributed to age-related differences in enzyme activity, potentially involving CYP3A4, leading to variations in drug metabolism, while the uniform CIP AUC observed among different age groups indicates that the overall levels of the active metabolite remain steady, despite variations in ENR plasma concentrations with age. In addition, the AUC_0-∞CIP_/AUC_0-∞ENR_ ratio was found to be higher in 1-week-old calves (33%) than in 1-day-old (18%) and premature calves (19%) [5,14]. This difference may be due to low enzyme activity in newborn and premature calves [36].

The antibacterial efficacy of ENR is dependent on concentration, with the AUC/MIC and C_max_/MIC pharmacokinetic/pharmacodynamic indices employed to assess its effectiveness [19,42]. The MIC of ENR for bacteria such as *Escherichia coli*, *Mannheimia haemolytica*, *Mycoplasma bovis*, and *Staphylococcus aureus* isolated from cattle was 0.02 μg/mL, 0.16–0.5 μg/mL, 0.25 μg/mL, and 0.13 μg/mL, respectively [43,44,45,46]. For effective treatment, the ƒAUC/MIC value is desired to be ≥125 for Gram-negative bacteria and ≥50 for Gram-positive bacteria [47]. An ƒAUC/MIC ratio of ≥125 was achieved for bacteria with MIC values of ≤0.09, ≤0.14, and ≤0.15 μg/mL in one-, four-, and eight-month-old calves, respectively. The ƒAUC/MIC ratio ≥ 50 in one-, four-, and eight-month-old calves was obtained for bacteria with MIC values of ≤0.23, ≤0.35, and ≤0.38 μg/mL, respectively. However, since the free drug fraction was estimated from the literature rather than directly measured, and the MIC values were derived from heterogeneous sources, these PK/PD integrations should be considered primarily as an orienting framework rather than definitive dose optimization guidelines. Within this theoretical context, it was observed that ENR could potentially be effective against bacteria with higher MIC values in 4-month-old and 8-month-old calves compared to 1-month-old calves.

This study has several limitations that impact the applicability of its results. It did not evaluate age-related changes in ENR plasma protein binding or measure hepatic enzyme activities and renal function directly, limiting insights into underlying mechanisms. Furthermore, the failure to assess age-related physiological changes hinders our understanding of how development influences pharmacokinetics. Only healthy animals were included, and a single IV dose (10 mg/kg) was used, so effects in diseased animals, other doses or routes, and multiple administrations remain unclear. Tissue distribution or clinical efficacy was not assessed. The calves used in this study showed significant differences in body weight, housing conditions, and nutrition. Therefore, housing conditions, nutritional differences, or different physiological conditions may have partially contributed to the age-related differences observed. To better understand these PK differences, future studies should include diverse populations, multiple dosing regimens, different nutritional strategies, enzyme and renal function tests, and PK/PD evaluations in diseased animals.

## 5. Conclusions

This study confirms that the pharmacokinetics of ENR and CIP at a dose of 10 mg/kg are significantly influenced by age in cattle. Younger animals exhibit higher metabolic conversion ratios and distinct pharmacokinetic profiles, including variations in volume of distribution, clearance, and half-life, compared to older calves. The observed differences suggest that age-related physiological changes impact the disposition of ENR and its metabolite, underscoring the importance of considering developmental stage when evaluating pharmacokinetic parameters. These findings contribute to a greater understanding of how growth and maturation modify drug behavior in cattle, highlighting the necessity for further pharmacokinetic and pharmacodynamic investigations to optimize dosing strategies and improve the overall efficiency of antimicrobial therapy in cattle of varying ages.

## Figures and Tables

**Table 1:** Pharmacokinetic parameters of enrofloxacin and ciprofloxacin following intravenous administrations of enrofloxacin (10 mg/kg) in one-, four- and eight-month-old calves (n = 6).

Parameters	1-Month Old	4-Months Old	8-Months Old
**Enrofloxacin**			
t_1/2ʎz_ (h)	2.52 (2.39–2.65) ^c^	3.36 (3.26–3.47) ^a^	3.03 (2.87–3.30) ^b^
AUC_0-last_ (h*µg/mL)	23.36 (21.05–26.11) ^b^	34.53 (30.19–40.37) ^a^	38.29 (33.70–43.07) ^a^
AUC_0-∞_ (h*µg/mL)	23.53 (21.20–26.30) ^b^	34.82 (30.54–40.72) ^a^	38.48 (33.88–43.25) ^a^
ƒAUC_0-last_ (h*µg/mL)	11.68 (10.52–13.05) ^b^	17.26 (15.10–20.19) ^a^	19.14 (16.85–21.53) ^a^
AUC_extrap_ (%)	0.73 (0.58–0.87)	0.81 (0.62–1.16)	0.50 (0.42–0.66)
MRT_0-∞_ (h)	3.39 (3.16–3.73) ^b^	4.42 (4.16–4.81) ^a^	3.39 (3.17–3.72) ^b^
Cl_T_ (L/h/kg)	0.42 (0.38–0.47) ^a^	0.29 (0.27–0.33) ^b^	0.26 (0.23–0.30) ^b^
V_darea_ (L/kg)	1.54 (1.41–1.72) ^a^	1.39 (1.18–1.64) ^a^	1.14 (1.00–1.30) ^b^
V_dss_ (L/kg)	1.44 (1.30–1.55) ^a^	1.27 (1.09–1.38) ^b^	0.88 (0.77–0.96) ^c^
**Ciprofloxacin**			
AUC_0-18_ (h*µg/mL)	9.99 (8.38–12.61)	8.74 (8.42–9.05)	8.86 (7.11–9.89)
AUC_0-∞_ (h*µg/mL)	10.21 (8.58–12.85)	8.96 (8.62–9.30)	9.05 (7.32–10.06)
AUC_extrap_ (%)	2.13 (1.72–2.58)	2.39 (1.83–2.89)	2.13 (1.73–2.85)
C_max_ (µg/mL)	1.44 (1.08–1.95) ^ab^	1.30 (1.21–1.44) ^b^	1.72 (1.33–2.38) ^a^
T_max_ (h)	2 (2.00–2.00) ^a^	1.00 (1.00–2.00) ^b^	1.00 (0.75–2.00) ^b^
AUC_0-∞CIP_/AUC_0-∞ENR_ (%)	43.39 (40.48–52.77) ^a^	25.73 (22.40–28.23) ^b^	23.04 (21.15–26.65) ^b^

Note: Data are presented as the geometric mean (min–max), except for T_max_, which is displayed as the median (min–max). ^a,b,c^: Varied characters in the same row are statistically different (*p* < 0.05). t_1/2ʎz_, elimination half-life; AUC, area under the concentration–time curve; AUC_extrap%_, area under the plasma concentration–time curve extrapolated from t_last_ to ∞ in % of the total AUC; MRT, mean residence time; Cl_T_, total body clearance; ƒ, calculated from the free enrofloxacin concentration; V_darea_, apparent volume of distribution; V_dss_, volume of distribution at steady state; C_max_, peak plasma concentration; T_max_, time to reach the C_max_.

## Data Availability

The original contributions presented in this study are included in the article. Further inquiries can be directed to the corresponding author.
